# The Canadian 24-Hour Movement Guidelines for Preschoolers With Disabilities: Occupational Therapists’ Perspectives

**DOI:** 10.1177/00084174251363008

**Published:** 2025-08-01

**Authors:** Leah G. Taylor, Aidan Loh, Liliana Alvarez, Kelly P. Arbour-Nicitopoulos, Shauna M. Burke, Patricia Tucker

**Keywords:** Disability, Movement, Occupational therapy, Young Children, Ergothérapie, incapacité, jeunes enfants, mouvement

## Abstract

**Background.** This study explores occupational therapists’ (OTs) perceptions of using the Canadian 24-Hour Movement Guidelines for the Early Years in their practice with preschool-aged clients (aged 3–4 years) with developmental disabilities. **Methods.** This study captured perspectives of the guidelines from OTs working in Canada via in-depth, virtual interviews (*n* = 11). Applied, deductive thematic analysis situated in the knowledge, attitudes, behavior framework, which provides a structured approach to understanding and addressing healthcare practitioners’ perspectives on implementing guidelines in practice, was conducted. **Findings.** Eight themes were identified: awareness and familiarity; agreement; outcome expectancy; self-efficacy; motivation; external—clients; external—guideline use; and external—environment. The results illustrate that while OTs may be motivated to incorporate the guidelines, concerns regarding their rigidity and applicability to diverse client needs hinder application in practice. **Conclusion.** The study highlights the need for additional resources and training to enhance OTs’ ability to adapt and apply the guidelines effectively, ensuring they meet the individual needs of children with developmental disabilities. This research underscores the potential for OTs to play a crucial role in promoting healthful movement behaviors in early childhood through the tailored application of the guidelines.

## Résumé

**Description.** Cette étude explore les perceptions des ergothérapeutes quant à l’utilisation des Directives canadiennes en matière de mouvement sur 24 heures pour la petite enfance dans leur pratique auprès d’une clientèle d’âge préscolaire (3–4 ans) ayant des déficiences développementales. **Méthodologie.** Cette étude a permis de saisir les perspectives des ergothérapeutes travaillant au Canada par le biais d’entrevues virtuelles approfondies (*n* = 11). Une analyse thématique appliquée et déductive située dans le cadre Connaissances, Attitudes et Comportements, qui fournit une approche structurée pour comprendre et aborder les points de vue des prestataires de soins de santé sur la mise en œuvre des directives dans la pratique, a été menée. **Résultats.** Huit thèmes ont été identifiés : sensibilisation et familiarité; accord; résultats espérés; auto-efficacité; motivation; externe – clientèle; externe – utilisation des directives; externe – environnement. Les résultats montrent que, même si les ergothérapeutes peuvent être motivés à incorporer les directives, les préoccupations concernant leur rigidité et leur applicabilité aux besoins diversifiés de la clientèle entravent leur application dans la pratique. **Conclusion.** L’étude souligne le besoin de ressources et de formations supplémentaires pour améliorer la capacité des ergothérapeutes à adapter et appliquer efficacement les directives, en veillant à ce qu’elles répondent aux besoins individuels des enfants ayant des déficiences développementales. Cette recherche souligne le potentiel, pour les ergothérapeutes, de jouer un rôle crucial dans la promotion de comportements sains à l’égard du mouvement dans la petite enfance grâce à l’application et à l’adaptation des directives.

## Introduction

Preschoolers (3–4 years) experience rapid growth and development, making engagement in health supporting behaviors during this life stage critical for overall well-being (Berk, 2018). Fostering physical activity, minimizing sedentary time, and encouraging healthy sleep habits have been shown to support preschoolers’ development (Carson et al., 2017; Kuzik et al., 2022). The Canadian 24-Hour Movement Guidelines for the Early Years (herein referred to as the guidelines) provide recommendations for an optimal daily balance of these behaviors. Specifically, these guidelines suggest that preschoolers should: accumulate 180 min of physical activity per day (including 60 min of energetic play); not be restrained for more than 1 hr at a time; limit sedentary screen time to less than 1 hr per day; and get 10–13 hr of sleep (including naps) each day (Canadian Society for Exercise Physiology [CSEP], 2017; Tremblay et al., 2017). Additionally, the guidelines provide messaging that the “Whole Day Matters” which encourages engaging in balanced movement behaviors over the course of an entire 24-hr day, as these are important for indicators of health at all ages (Rollo et al., 2020).

Research indicates that children with developmental disabilities are less physically active, have shorter sleep patterns, and experience higher rates of sedentary time than children without disabilities, all of which have been associated with negative implications for development (Edgin et al., 2015; Ganz et al., 2020; Reynolds et al., 2019; Taylor et al., 2023). Developmental disabilities are lifelong impairments that can affect social, emotional, physical, or cognitive development (Zablotsky et al., 2019), and through environmental restrictions, limit full participation in society on an equal basis with others (United Nations, 2006). While the guidelines have been deemed appropriate for children with developmental disabilities, it is suggested they be implemented with guidance from a healthcare practitioner (CSEP, 2017). Moreover, parents have reported healthcare practitioners are trustworthy sources for communicating the guidelines (Riazi et al., 2017).

Occupational therapists (OTs) are well-positioned to facilitate the dissemination of the guidelines (Taylor et al., 2025). Specifically, OTs play a fundamental role in supporting preschoolers with developmental disabilities in play, movement, and sleep, which are all fundamental occupations of childhood (Case-Smith, 2013). Physical activity in the early years, typically in the form of active play (Truelove et al., 2017), is considered a primary occupation for children (Lynch & Moore, 2016). Similarly, adequate sleep provides a foundation for occupational engagement, and OTs can support changes to facilitate effective sleep routines (Piller et al., 2021). OTs also consider the balance of clients’ sedentary activities for leisure and learning pursuits which impact optimal health outcomes (Dwyer et al., 2009). Although some OTs already promote these three movements in practice (Hill et al., 2022; Piller et al., 2021; Taylor et al., 2024), research indicates that many OTs may lack the expertise to explain, modify, implement, and monitor adherence to the guidelines for pediatric therapeutic use (Handcock & Tattersall, 2012; Honaker & Meltzer, 2016; Piller et al., 2021; Taylor et al., 2025).

The knowledge, attitudes, behavior framework provides an approach to understanding and addressing healthcare practitioners’ perspectives on implementing guidelines in practice (Cabana et al., 1999). This framework suggests to effectively change healthcare provider behaviors, their knowledge and attitudes must be first addressed, but all three dimensions are thought to influence healthcare professionals’ implementation of guidelines (Cabana et al., 1999; Fischer et al., 2016). Knowledge focuses on practitioners’ awareness of the guidelines, and familiarity with the underlying evidence, which is shaped by training, experience, and access to relevant information. Attitudes, the second dimension, are shaped by the practitioners’ personal views, which encompass individual agreement with the guidelines, outcome expectancy, self-efficacy, and motivation to use the guidelines. Finally, behavior is the action of practitioners, which is influenced by external barrier sources: guideline-related (i.e., characteristics of the guideline that do not align with current practice), client-related (i.e., inability to reconcile the guideline with client preferences), and environmental-related (i.e., changes or factors outside of their control, including access to resources; Cabana et al., 1999; Fischer et al., 2016).

Despite the suitability of OTs to implement the guidelines with preschoolers with developmental disabilities due to their focus on promoting engagement in these relevant childhood occupations, the guidelines are underutilized by practitioners who work with this population, and OTs lack confidence to apply them (Taylor et al., 2025). Examining OTs’ knowledge, attitudes, and behaviors related to employing the guidelines is essential to understanding perspectives, and ideally, to enhancing guideline implementation with young children in the future. As such, the purpose of this study was to explore OTs’ perceptions regarding the use of the guidelines, in a Canadian context, in their practice with preschoolers with developmental disabilities. Understanding OTs’ insights will identify if resources are needed to support guideline application in practice with this population.

## Method

This study was conducted as part of a larger project which aimed to explore the application of the guidelines as an occupational therapy approach for preschoolers with developmental disabilities (Taylor et al., 2025). The present study sought to gain a deeper understanding of OTs’ perceptions of incorporating the guidelines in their practice, by way of semistructured, in-depth interviews. The primary author is a PhD candidate and occupational therapy student who identifies as a White, able-bodied woman with professional experience in adaptive sports and inclusive community programming. These intersecting experiences shape her reflexive practice and the ways in which data is analyzed in this study. This study was informed by a critical realist paradigm. This stance acknowledges that while there is a reality independent of our perceptions, our understanding of it is always mediated by social, cultural, and historical contexts (Lawani, 2021). Ethical approval for the study protocol and all related documents was obtained from the Office of Research Ethics at Western University (REB #121341).

### Recruitment and Participants

OTs from across Canada who were originally recruited to participate in an online questionnaire (*n* = 28; Taylor et al., 2025) were invited to participate in a single one-on-one interview. A detailed account of recruitment for the larger study is available elsewhere (Taylor et al., 2025). Briefly, research estimating that approximately 10–12 interviews are required to reach saturation guided the sample size recruitment (Hennink & Kaiser, 2022). A subsample of 11 OTs indicated interest. Participants were eligible to participate in the interview if, in addition to the original eligibility criteria (e.g., registered with a Canadian occupational therapy regulatory body, had experience working with children aged 3–4 years with developmental disabilities, and had internet access), they: (a) spoke English; (b) were willing to conduct an interview on a virtual platform; and (c) agreed to have the interview audio recorded. All 11 interested OTs met the criteria, were invited to participate, and agreed to do so. Informed consent was obtained from all participants prior to data collection.

### Data Collection

Interviews took place from February to March 2023 (see Table 1) were developed by the research team, based on the knowledge, attitudes, behavior framework (Cabana et al., 1999). Participants were sent the 24-Hour Movement Guidelines for the Early Years (CSEP, 2017) prior to their interview to review. The resource was also shared on the screen throughout the interview. Interviews lasted ∼45 min and participants were able to skip questions or end the interview at any time. Interviews were facilitated on Microsoft Teams, and audio recorded and transcribed in real time by this software.

**Table 1:** Interview Questions Integrated into Knowledge, Attitudes, Behavior Framework and Structural Coding.

Dimension of framework	Structural themes	Code identifier	Interview questions
Knowledge	Awareness and familiarity	Were clinicians aware of their existence? Did they know the guidelines existed? Have they previously used the guidelines? What is their self-reported level of familiarity on the guideline?	Prior to completing our survey, were you aware of the 24-Hour Movement Guidelines for the Early Years? In what contexts have you seen the guidelines previously? **OR** Is this something you’ve heard about outside of the early years context?
Attitudes	Agreement	Do they think it's relevant and has a role in OT? Do they see benefits for their clients? Is it applicable to their clients? Are guidelines well-delivered or oversimplified? Does the guideline reduce autonomy?	What are the benefits to applying these guidelines in your practice? What do you like about them? Can you give an example of a time you may have integrated these behaviors? What are the challenges to incorporating these guidelines into practice? Tell me your thoughts on the ease/convenience of using the guidelines? What makes them difficult to use?
	Outcome expectancy	The expectation that a given behavior will lead to a particular consequence. If a clinician believes that a recommendation will not lead to an improved outcome, the clinician will be less likely to adhere/employ. An important reason for clinician nonadherence is a belief that they will not succeed.	In your opinion, how could the integration of these guidelines align with your patients’ goals? Can you think of an example of how you could integrate these behaviors into your typical care plans? **OR** Can you expand on why you feel the guidelines are irrelevant to the goals of your clients? Provide an example/hypothetical if possible.
	Self-efficacy	Self-efficacy is the belief that one can actually perform a behavior. It influences whether a behavior will be initiated and sustained despite poor outcomes. Low self-efficacy due to a lack of confidence in ability or a lack of preparation may lead to poor adherence.	Can you describe your confidence to incorporate the guidelines into your practice? Can you describe how capable you feel of modifying the recommendations for application with different client needs? **OR** What do you think is limiting your confidence? What do you feel like you would need to improve your level of confidence?
	Motivation	Is this guideline something the clinician is motivated to use? Why or why not?	Describe your level of motivation to incorporate the guidelines into your practice. How would this benefit your clients? **OR** What's holding you back?
Behavior	External—clients	The inability to reconcile client preferences with guideline recommendations. Clients may be resistant or perceive no need for guideline recommendations. In addition, a client may perceive the recommendation as offensive or embarrassing.	*No specific questions provided. Probed through follow-up questions to “outcome expectancy: question.*
	External—guideline use	What guideline characteristics make the clinician more/less likely to implement? Are the guidelines convenient or easy to use? For example, elimination of an established behavior may be more difficult to follow than guidelines that recommend adding a new behavior.	Describe your access to resources to incorporate the guidelines in practice. This could include (e.g.) time, training, or tools, or support from your organization. What are the resources you find most useful? What resources would you need to be in place to use/consider using the guidelines? Why are those resources important? Would there be a beneficial resource for supporting integration into practice in your opinion?
	External—environment	Changes not under clinician control, such as acquisition of new resources or facilities.	

*Source:* Adapted from Cabana et al. (1999).

### Data Analysis

Procedures for applied deductive thematic analysis provided by Guest et al. (2014) were used to analyze the data. The first author listened to the recorded interviews, cleaned and anonymized the interview transcripts, and created a code book aligning with the knowledge, attitudes, behavior framework definitions (see Table 1). All subsequent coding and analysis were conducted in QSR NVivo (version 12). In the first round of coding, two researchers used deductive analysis to parcel the transcription into the eight structural theme categories from the knowledge, attitudes, behavior framework (Table 1). The coders analyzed the data separately and met for weekly reliability checks after every three transcripts to evaluate intercoder reliability, through discussion and consensus on a combined coding document (Creswell & Clark, 2017). The second round of coding was conducted by the first author to gather similar concepts and identify trends within each structural theme (Guest et al., 2014). The second researcher who had knowledge of the study but whose involvement was limited to coding the interviews reviewed the codes to address confirmation bias. All authors then reviewed the codes and the associated quotes to ensure trustworthiness (Anney, 2014). Throughout the analysis, measures were taken to ensure rigor and trustworthiness of the analysis regarding credibility, confirmability, dependability, and transferability (Anney, 2014; Guba & Lincoln, 1989). This included: peer debriefing (i.e., the primary author met regularly with the secondary author not involved in data collection to discuss analytic decisions and challenge interpretations, helping to prevent bias and strengthen the authenticity of findings); reflexive journaling (i.e., throughout data collection and analysis, the research team maintained notes on decisions and changes to methods over time, evolving assumptions, and positionality, to document the evolution of the analysis); and negative case analysis (i.e., the researchers actively sought out and incorporated perspectives that diverged from dominant themes, ensuring findings reflected the full complexity of participant experiences rather than a singular narrative).

## Results

Participants (*N* = 11) worked in four provinces (British Columbia [BC; *n* = 2], Alberta [AB; *n* = 3], Saskatchewan [SK; *n* = 2], and Ontario [ON; *n* = 4]), and all identified as being a woman. All participants had worked as an OT in pediatrics for over 5 years, with an average of 20 years of experience (*SD* = 8.6; Range = 5–36 years). Quotes are presented with years of practice in pediatrics and the alpha code for province.

### Knowledge

#### Awareness and familiarity

Most participants indicated they were not previously aware of the guidelines. For those who were somewhat aware, this was outside of the early years cohort, and most often referred recommendations for sleep for older children (5–17 years). Participants also discussed that they were somewhat familiar with the movement concepts and health outcomes, especially in the context of occupations. However, most were not aware of durations and recommendations associated with each behavior. For example, Participant 2 (5 years; ON) indicated: “*I guess I knew guidelines existed. I don't think I ever referred to the exact hours. It was more what are your goals and encouraging kind of appropriate activities for the certain kids or whatever?.*”

Despite lacking awareness on the guidelines specifically, several participants were familiar with and possessed knowledge on the benefits of engagement in movement generally, and incorporated physical activity, sedentary time, and sleep into their practices in preschoolers with developmental disabilities. Once introduced, some participants implemented the recommendations in practice. This was demonstrated, for example, by Participant 6 (22 years; AB): “*It was a good refresher and for instance, [a parent] told me their child was getting a 10-h sleep and I was very* quickly *able to say that's good. I didn't have to think, oh, is that the right amount?.*”

### Attitudes

#### Agreement

Overall, participants agreed the guidelines were generally relevant to their role as OTs and felt it was their responsibility to advocate for use of the recommendations in the everyday environments of preschoolers with developmental disabilities (i.e., home, childcare settings). They also felt there were opportunities to integrate the guidelines in practice as relevant occupations of preschool-aged children with developmental disabilities, and to translate and adapt the recommendations to educate caregivers. For some participants, the guidelines aligned with their professional philosophies, were well-suited to their current practice, and validated and reaffirmed current clinical strategies. For example, Participant 7 (30 years, ON) identified the alignment: “*They're clear. They're simple. They're reinforcing all the other recommendations that myself and the colleagues that I work with in a multidisciplinary team are making anyway. So, it's just a breath of fresh air to be able to refer to them*.”

The major criticism on guideline use came from the rigidity of the recommendations, and the implications on clients with a spectrum of disabilities. For example, the recommendation of limiting sedentary screen time to less than 1 hr per day was a point of contention for some respondents, who felt this was unfair to caregivers who used screen time for personal respite, or to engage in their own occupations (e.g., home management). Moreover, participants expressed concern that the guidelines were not developed with the varying needs of children with developmental disabilities in mind. Instead, many participants noted that it was their role, as OTs, to think about the recommendations as targets, but to also be able to adapt the guidelines to each client's goals, needs and preferences. Participant 1 (17 years; BC) phrased this as: “*How do we start to make changes to that movement pattern versus categorizing things by numbers?*.”

Participants pointed to a requirement for a high level of autonomy necessary to scaffold the guidelines with the individual in mind, and to adapt recommendations. Participant 8 (13 years; SK) provided insight into this area of concern:

*With this being the target or the gold standard that we're trying to hit, knowing the children that I work with, this could be very hard or look very different than … with a child that doesn't have some type of disability … While this might be the goal, we would have to work really hard to make a lot of adjustments for some kids to make this possible.*

Due to the oversimplified nature of the current format and the level of autonomy required to adapt the guidelines, participants had concerns regarding balancing using the resource and addressing individual client needs.

#### Outcome expectancy

Most of the participants felt that using the guidelines would support positive client outcomes, and as Participant 4 (30+ years; ON) put it, movement is “*the lens that I use to help with human occupation*.” Positively affecting children's well-being was one identified outcome of integrating the guidelines into practice. This included supporting mindfulness and positive mood, finding calm, and focusing on stress management and anxiety reduction when working with children with disabilities.

Several participants also referred to the potential benefits of the guidelines in supporting self-regulation, including each movement behavior individually and in tandem for preschoolers with developmental disabilities. Participants felt that the outcomes afforded by engagement in movement were the ability to partake in occupations such as learning, play, and socializing. Participants spoke to their current implementation of physical activity as a preparatory and calming activity for children with some types of disabilities (e.g., autism spectrum disorder, attention-deficit/hyperactivity disorder). Participant 3 *(13 years; ON)* described the specific physical activity-related benefits they observed in their clients: “*Attention and concentration, especially in the ADHD population. If those kids are given the opportunity to move enough, their focus, their attention and concentration during the seated activities becomes more purposeful. So that's for me the direct benefit.”*

Other participants also suggested that physical activity helped clients be available for organized sitting activities such as reading and storytelling, to support transitions in childcare, and to expend energy to support sleep. Good quality sleep was also acknowledged as crucial to children's regulation for playing or learning in childcare settings. Moreover, the dysregulation potential of screens was acknowledged by participants, who felt it was their role to discuss the importance of limiting screen time for preschoolers with developmental disabilities to reduce negative impacts on self-regulation, play and sleep, and daily routines.

Some participants expressed concern that the guidelines would not lead to positive outcomes for their clients’ well-being, due to contextual factors influencing movement for preschoolers with disabilities. Examples provided included unsupportive environments, underlying concomitant medical issues and co-occurring behaviors, family contexts, and client preferences. Some participants expressed that for these reasons, they might be less likely to employ the guidelines to avoid overwhelming caregivers. Participant 9 (36 years; AB) provided an anecdote explaining this situation:

*I have one little guy right now for example, who's 4, he's got autism. He goes to sleep okay, but he wakes up at like 4 in the morning. He's up until about 7, then falls back asleep for a few hours …* *Trying to change the sleep routines are really challenging because … he's also a super picky eater…* *So, he's not even getting enough nutrition to sustain him to sleep all night. So, until we resolve the picky eating, I don’t think we’re going to make a difference on the sleep, right? …* *But sometimes there's more to it than just sleep hygiene.*

#### Self-efficacy

Participants’ self-reported levels of self-efficacy to integrate the guidelines into practice were mixed. Some participants were unsure of how they would use the current resource with prescriptive times allocated to each activity, while also balancing caregiver expectations and competing professional responsibilities. For example, Participant 6 (22 years; AB) indicated:

*I definitely see* challenges *and would appreciate formats and ways of helping to organize thoughts around these things. And definitely I think that there would have to be modification … So, I think that is an obstacle. When I'm just racing out the door to get to the next client, I am unable to do that breakdown for the client. I'm not confident that I have a way of doing that?*

On the other hand, some participants felt they were generally confident to incorporate the guidelines if they could use them flexibly to meet the client's needs. Overall, participants reported higher confidence to use the physical activity and sedentary time recommendations than the sleep recommendation. This confidence did not always extend to action, in that some clinicians felt they needed more concrete strategies to support integration of the guidelines.

#### Motivation

Participants’ motivation levels for guideline use were mixed. Participants’ negative motivation primarily stemmed from concerns with the family context and the risk of overwhelming caregivers by using the recommendations prescriptively. Participant 10 (19 years; AB) provided an example of this concern:

*Some [parents] are just very energetic and on top of things and others, they’ve got so many things* going *on and no judgment that's just sometimes where life is at. And so, to give them something more … that might not be beneficial. Or could be discouraging for some too, right?*

Participants sharing this concern suggested that movement might not be the family's biggest priority. Participants felt it was their role to reaffirm and encourage caregivers, and to acknowledge that incorporating these behaviors can be difficult.

Participants’ positive motivation to use the guidelines stemmed from two main areas. The first was the holistic, evidence-based messaging of the *whole day matters*. For some, such as Participant 7 (30 years; ON), this motivation stemmed from their perspective on their professional responsibilities: “*People are being too concrete when they look at movement guidelines and they think that that has to do with physio or exercise or sports. When we need to shift the lens to look at how* movement *impacts wellness … And these are huge OT roles*.”

The second area was the alignment of the guidelines to support their current care programs and seeing an opportunity to use the recommendations as an educational resource. Participants were motivated to organize and translate to caregivers the importance of a daily balance of meaningful movement. Participant 8 (13 years; SK) discussed how they would incorporate the guidelines flexibly, while tailoring them to individual clients:

*I kind of like the three categories and often when I am working with children … I do kind of categorize information in the like, how is the* sleep*? …* *And then what are you guys doing for fun? Like, fun for kids is moving and playing … And then you know, what does the sit category look like for them? … How long do they spend on the on the TV or on a screen? And what's your travel needs like? … And what kind of interactive activities are you guys doing with where you are sitting a little bit more but there's still like that rich connection piece happening. So, I do feel like I kind of organize things within these categories, but just never said, here's this guideline that's already created. We kind of forge a new one for every family but always considering these categories.*

### Behavior

#### External—Clients

Clients’ perspectives, needs, and preferences seemed to be the biggest barriers to practitioners’ use of the guidelines. Participants were acutely aware of individual needs and preferences of each child when considering the use of the recommendations, and potential challenges that may arise due to varying abilities or limitations. Participant 10 (19 years; AB) provided an example:

*For some kids, depending on if they have different diagnosis or what their developmental stage is at … It may not be realistic to move for 180 min a day. So, what can that look like? Say, if they are in a wheelchair, or* something *like that. What kind of activity could that look like for them … And what kind of things can we do for them so that they are moving and engaging in the world around them?*

In addition to the child's individual needs, participants were also concerned about caregivers’ capacity to implement the guidelines. While some families were noted to excel with structure and homework, participants were concerned about placing additional demand on families already undergoing stress. They felt generally, caregivers understand the importance of movement, but it can be a challenge to execute the recommendations because of personal situations (e.g., caregivers experiencing disability themselves or financial status), everyday responsibilities, skills to execute, and unsupportive external environments. Participants were wary about asking families to take on additional tasks when movement was not already a part of their routines, as this could introduce imbalances and caregiver burden. Participants also emphasized the need for resources for families if the guidelines were to be implemented in their care plans. This included identifying barriers and individual family supports through physical, financial, and/or community resources.

#### External—Guideline use

Participants indicated they would be less likely to incorporate the guidelines should they be required to use the durations associated with each recommendation, as they were too prescriptive. However, participants also felt that the content of the guidelines fit well with the focus of their practices, so they could be flexibly incorporated. For example, reframing the recommendations from durations to goals and activities in the client's day. Participant 1 (17 years; BC) provided an example: “*I see how you set up the guidelines is by quantitative numbers. And so, for me it's not so much about. Let's aim to get 100 or 60 or 5 min of play time, it's like can we engage your kid … how can we incorporate movement in their day-to-day?*”

Overall, participants generally felt the layout was easy, convenient and a useful way to consider the child's daily occupations and to break up the daily routine. As a reference tool, whether printed or just mentioned, participants felt that they could flexibly integrate the guidelines in their care approaches. Participant 11 (16 years; BC) who, since introduction to the resource by the research team was using the guidelines in practice, provided an example of the need for flexibility:

*This is obviously a day-to-day approach, more in the absence of the therapist and not during the session. I have started giving this as a* handout *to parents as well, so that they can self-check and self-monitor what's happening and if they're seeing changes in behaviour or routines, they can quickly reflect back to see … It's there as a rough guideline, but not a set-in stone rule to follow*.

#### External—Environment

Three main environmental facilitators which supported use of the guidelines were identified by participants. The first was professional development opportunities provided on movement. This most often had to do with sleep and the occupations of preschoolers. The second was having high-quality, external resources to access, typically retrieved from children's hospitals and university research centers. The third was networks of colleagues to share ideas and resources. This included other OTs, special interest groups, and the allied health team (e.g., physiotherapists, recreation therapists, doctors).

While professional development opportunities were identified as a facilitator, the lack of high-quality training on incorporating movement through the lens of childhood development, especially in preservice learning, was identified as a barrier to incorporating the resource. For example, Participant 4 (36 years; ON) who is a course instructor for an occupational therapy program provided the following insight:

*What I'm seeing is that this would be an important foundational thing to be taught and trained. So that people know that this is very important for human development at this age … I don't know if it's enough for me to talk about it for five minutes and for that to be translated, that's the big question mark*.

Participants offered suggestions on opportunities to support practice. The most common was knowledge translation materials for practice implementation. Most participants suggested an occupational therapy specific resource and subsequent training, with a focus on best practices for incorporating the guidelines with case studies. Participant 11 (16 years; BC) emphasized the need for in-depth training opportunities to support implementation in practice:

*The real-life examples like case studies during training would be beneficial. And then a platform for I've now tried technique* X, Y *and Z. It's not working. Do I need to keep tweaking it? Do I need to abandon ship and try something else? The clinical judgment for that does get tricky*.

Participants wanted to pool resources, share examples and success stories, and have hands on training to incorporate the guidelines in practice. Some suggestions for learning environments included webinars, mentorship with colleagues, e-learning modules, community of practice seminars, and preservice learning.

## Discussion

The purpose of this study was to explore OTs’ perceptions regarding the use of the guidelines in practice with preschoolers with developmental disabilities in Canada. This study was designed in line with the knowledge, attitudes, behavior framework to examine influences on guideline use and uptake in practice (Cabana et al., 1999). Participants discussed facilitators and barriers that shape guideline use in practice. These perspectives provide insight into understanding applications of the guidelines within occupational therapy, and aligning dissemination efforts to preferences and needs of OTs (Faulkner et al., 2016).

Healthcare practitioners have been identified as key disseminators of the guidelines, supporting client behaviors through assessment, counseling behavior change, prescribing, and referring (Fowles et al., 2018). OTs in Canada have indicated they feel that the guidelines align closely with their role of supporting preschoolers’ occupations (e.g., play and sleep), and generally, the four roles of practitioners suggested by Fowles et al. (2018) may apply to the way OTs can facilitate movement in practice (Taylor et al., 2025). However, this study demonstrated that capacity building supports are necessary to increase guideline use, and this must be matched with flexibility of application for clients.

This research illustrated that the current guidelines do not align with how OTs work with clients. Participants felt that movement is important, independent of duration. As well, OTs felt that the simplified daily time recommendations were too prescriptive, while not considering the various abilities, contextual factors, and needs of children with developmental disabilities. These perspectives align with previous global attention to the value of promoting movement while being cognizant of the specific needs of children with disabilities in movement behavior participation (Sit et al., 2022). OTs in this study felt that the recommendations should be restructured as flexible targets for preschoolers with developmental disabilities, in support of goal achievement. This would offer adaptable, yet achievable targets to work toward. OTs also felt that strategies to make the guidelines person-centered, considering contextual factors influencing movement for preschoolers with developmental disabilities were necessary. The results suggest that to support use of the guidelines, an implementation resource specific to OTs would be valuable. Globally, this type of resource is not yet available to support use of the guidelines for OTs practicing with any client population.

Regarding guideline content, OTs both within this study and previous studies, generally agree that the movement recommendations fall within their scope (Hill et al., 2022; Piller et al., 2021; Taylor et al., 2025). However, participants in this study were not completely confident they possess the knowledge and skills to integrate the recommendations in practice. Low self-efficacy to incorporate movement in practice has been attributed to a lack of relevant and necessary training (Albert et al., 2020). The findings of this study reiterate the importance of contextualizing the guidelines within occupational therapy, and to establish effective training to improve self-efficacy. This could include building knowledge and readiness to incorporate movements in practice via professional development opportunities and knowledge translation resources (Jones et al., 2019).

This research is not without limitations. First, social desirability bias could have affected the findings as information was collected via interviews. As well, given participants were recruited from a subsample of our previous study (Taylor et al., 2025), this may have also led to sampling bias. Interviews were only conducted in English, which may have discouraged/excluded French-first language OTs from participating. Finally, all participants self-identified as women, had worked as an OT in pediatrics for over 5 years (with an average of 20 years of experience), and all lived in one of four provinces and territories. This means that diverse perspectives (including more junior practitioners) may have been missed; however, information available through the Canadian Institute for Health Information (2022) suggest that our small sample was somewhat representative of current OTs in Canada, based on province of practice and gender. Future research with larger samples and early-career clinicians or recent graduates would improve the transferability of these results.

## Future Recommendations

Participants highlighted the potential value of the guidelines in occupational therapy. However, OTs recommended development of an implementation resource supportive of guideline integration specific to practice with preschoolers with developmental disabilities. Based on the interpretation of findings from this study, the authors have identified five recommendations for the development of a future implementation tool.

1. Therapeutic activities occur within and outside of therapy, such as in the home or childcare settings, where OTs are not present. An implementation resource should be both client and clinician facing (Faulkner et al., 2016), and connect users to high-quality, external resources supportive of goals.
2. An implementation resource should support individual guideline tailoring. The interaction between a child's disability diagnosis, their environment, and the task at hand may influence the types and intensities of movement that children can engage in (Taylor et al., 2023). Therefore, this resource must serve to modify the guidelines and adapt activities to support client-centered goals.
3. Engaging preschoolers in meaningful movement on their own terms was prioritized by OTs in this study as a necessity. An implementation resource should draw on the “Whole Day Matters” messaging (CSEP, 2017). Specifically, combined movement across the day has important implications for preschoolers’ well-being (Rollo et al., 2020). This perspective could be used to reframe the guidelines as flexible targets, versus rigid rules of engagement.
4. Practitioners identified the need to provide outcome-focused evidence of integrating movement in day-to-day routines to support user-friendly knowledge translation for families.
5. OTs identified that time and competing priorities were barriers to the incorporation of new resources into already busy appointments. Resources should be easy to administer. Research demonstrates this is likely to increase the uptake of implementation tools by practitioners (Morgan et al., 2023).

## Conclusion

As healthcare practitioners, OTs are well positioned to support uptake of the guidelines for preschoolers with developmental disabilities. This research suggests that while the guidelines are relevant, more work is needed to address the appropriateness of incorporating the resource in practice. Specifically, training should address OTs’ self-efficacy to implement the guidelines and contextualize movement within occupational therapy to support well-being. Such training could build practitioners’ knowledge and readiness to incorporate movement. Moreover, for OTs to utilize the guidelines in practice, an implementation resource with a person-focused approach would be an asset. This would be an important step in supporting integration of the guidelines and their associated benefits to wellbeing in the daily lives of preschool-aged children with developmental disabilities.

## Key Messages

- The Canadian 24-Hour Movement Guidelines for the Early Years are relevant to occupational therapy practice with preschoolers with developmental disabilities.
- Occupational therapists require tailored professional development and resources to effectively apply these guidelines in client-centered practice.
- A future implementation resource should be client and clinician facing; support individual tailoring; draw on the “Whole Day Matters”; contextualize the guidelines with relevant outcomes; and, be quick and easy to administer.
